# A Genome-Wide Association Study Identifying Genetic Variants Associated with Growth, Carcass and Meat Quality Traits in Rabbits

**DOI:** 10.3390/ani10061068

**Published:** 2020-06-20

**Authors:** Xue Yang, Feilong Deng, Zhoulin Wu, Shi-Yi Chen, Yu Shi, Xianbo Jia, Shenqiang Hu, Jie Wang, Wei Cao, Song-Jia Lai

**Affiliations:** 1 Farm Animal Genetic Resources Exploration and Innovation Key Laboratory of Sichuan Province, Sichuan Agricultural University, Chengdu 611130, China; yangxue790702@163.com (X.Y.); fdeng@uark.edu (F.D.); wzlneil@163.com (Z.W.); sychensau@gmail.com (S.-Y.C.); 18227551690@163.com (Y.S.); jaxb369@sicau.edu.cn (X.J.); sqhu2011@163.com (S.H.); wjie68@163.com (J.W.); caowei_2005@126.com (W.C.); 2 Chengdu Academy of Agriculture and Forestry Sciences, Chengdu 611130, China; 3 Special Key Laboratory of Microbial Resources and Drug Development, Research Center for Medicine and Biology, Zunyi Medical University, Zunyi 563000, China

**Keywords:** *Oryctolagus cuniculus*, SNPs, SLAF-seq, genome-wide association study, growth trait

## Abstract

**Simple Summary:**

Rabbit meat has been widely consumed in China and is considered as an ideal food source due to its high protein, low fat, low cholesterol and low sodium contents. The growth rate, carcass characteristics and meat quality are considered economically important traits in the rabbit industry. Genomic selection (GS) could facilitate genetic selection for important economic traits, however, the lack of molecular markers for these traits limits the application of GS in rabbits. Genome-wide association study (GWAS) has the potential to comprehensively identify trait-associated molecular markers and has been applied in animal and plant research. In this study, GWAS was used to examine growth, carcass and meat quality traits of meat rabbits based on specific-locus amplified fragment sequencing (SLAF-seq) technology to identify significantly associated SNPs and functional genes, to be used as a basis for prompting the application of GS in rabbits.

**Abstract:**

Growth, carcass characteristics and meat quality are the most important traits used in the rabbit industry. Identification of the candidate markers and genes significantly associated with these traits will be beneficial in rabbit breeding. In this study, we enrolled 465 rabbits, including 16 male Californian rabbits and 17 female Kangda5 line rabbits as the parental generation, along with their offspring (232 male and 200 female), in a genome-wide association study (GWAS) based on SLAF-seq technology. Bodyweight at 35, 42, 49, 56, 63 and 70 d was recorded for growth traits; and slaughter liveweight (84 d) and dressing out percentage were measured as carcass traits; and cooking loss and drip loss were measured as meat quality traits. A total of 5,223,720 SLAF markers were obtained by digesting the rabbit genome using RsaI + EcoRV-HF^®^ restriction enzymes. After quality control, a subset of 317,503 annotated single-nucleotide polymorphisms (SNPs) was retained for subsequent analysis. A total of 28, 81 and 10 SNPs for growth, carcass and meat quality traits, respectively, were identified based on genome-wide significance (*p* < 3.16 × 10^−7^). Additionally, 16, 71 and 9 candidate genes were identified within 100 kb upstream or downstream of these SNPs. Further analysis is required to determine the biological roles of these candidate genes in determining rabbit growth, carcass traits and meat quality.

## 1. Introduction

Rabbit meat has a long history of consumption starting from around 1100 BC [1]. It has high nutritional value and is considered a healthy food because of its high protein content and low fat, cholesterol and sodium [2]. Growth rate, carcass characteristics and meat quality are considered important economic traits in rabbit breeding. In the past few years, researchers have worked on improving growth performance and meat quality by advanced molecular breeding methods. Dozens of single-nucleotide polymorphisms (SNPs) have been identified by resequencing gene regions and found to be associated with growth traits in rabbit. Melanocortin receptor 4 (*MC4R*) [3], fat mass and obesity-associated (*FTO*) [4,5], *LEP* [6], *TBC1D1* [4] and *GHR* [7] genes are correlated with growth and carcass traits in rabbit. However, these researchers mainly investigated the correlation between a single SNP present in a specific DNA fragment with a given trait using low-throughput methods [4,5,6]. For complex traits such as growth performance and meat quality, large-scale analysis is necessary to detect trait-associated SNPs.

Genome-wide association study (GWAS) [8] represents a powerful approach to correlating SNPs and functional genes with quantitative traits. SNPs associated with a specific trait can be considered as molecular markers for application in genomic selection (GS) [9] and as genetic markers [10]. The most important step in GWAS is to acquire high-quality SNPs at the genome-wide level. A high-density SNP array is a high-throughput, cost-effective genotyping assay and is the most widely used genotyping method in GWAS [11,12]. Although there are still disadvantages, for example, that only known SNPs can be detected, there are high costs and great effort involved in establishing an array and that marker distribution is biased [13], it has become possible for researchers to perform GWAS using 10,000 individuals. Whole-genome resequencing is another major genotyping method that has been used over the last 10 years. It is a powerful method for whole-genome SNP discovery [14,15]. However, whole-genome resequencing can be prohibitively expensive in GWAS. Therefore, specific-locus amplified fragment sequencing (SLAF-seq), a high-resolution strategy for large-scale genotyping at the genome level, is a great alternative approach to SNP genotyping in non-popular research species such as rabbits [16]. Compared with high-density SNP arrays and whole-genome resequencing, SLAF-seq is an efficient method for de novo SNP discovery with such advantages as high genotyping accuracy, relatively low cost and a high capacity for large sample sizes. SLAF-seq has been successfully applied in chicken [17,18] and pig [19].

Despite the great success of GWAS in animal science [20], including a recent GWAS study successfully performed by Sosa-Madrid and colleagues to identify genomic regions associated with the intramuscular fat of rabbits based on a high-density SNP array [21], there is still a lack of large-scale research studies linking important economic traits to candidate genes in rabbit. Identification of SNPs associated with economically important traits via GWAS, as a first step, would provide a basis for further improving the breeding efficiency of rabbits. Here, we performed a GWAS study of the growth, carcass and meat quality traits of meat rabbits based on SLAF-seq technology to identify the associated SNPs and to predict functional genes. This study will provide a molecular basis for marker-assisted selection and gene-based selection to improve those traits.

## 2. Materials and Methods 

### 2.1. Ethics Statement

All animal experiments were approved by the Institutional Animal Care and Use Committee of Sichuan Agricultural University (Permit Number: No. DKY-B20141401). The experiments were performed in accordance with the institutional regulations (no public availability). 

### 2.2. Animals and Phenotypes

For the experiment, 53 Californian female rabbits and 22 Kangda5 line rabbits, a commercial strain of meat rabbit from Qingdao Kangda Foodstuffs Co., Ltd., were selected as the parental generation to generate crossbred F1 offspring. Rabbits with the same day of mating (2 May 2017) and the same day of delivery (2 June 2017), including 16 male Californian rabbits and 17 female Kangda5 line rabbits, were enrolled in the study. All rabbits were fed a pellet diet (10.9 MJ/kg DE, 16.5% CP, 13.3% CF) and housed in cages of 50 cm × 40 cm × 40 cm in size. During day 35–70, two rabbits were placed into one cage block and one rabbit was raised in a separate cage block from 70 days until slaughter. An environmental conditioning control system was used when the conditions were out of the normal range with respect to the ventilation (0.5–4 m^3^/h/kg), temperature (18–25 °C) and humidity (20%–70%). A 12 h period of artificial normal environmental lighting was provided. All rabbits of the F1 generation were weaned at the age of 35 days.

Bodyweights were recorded on days 35, 42, 49, 56, 63 and 70. In China, meat rabbits are usually slaughtered at 70 or 84 days old. Therefore, we recorded bodyweight at day 70 and slaughtered at day 84. A total of 432 F1 generation rabbits, including 232 male and 200 female rabbits, was slaughtered by electrical shock after fasting for 24 h. The skin and head were separated from the body by cutting at the level of the third caudal vertebra and of the distal epiphyses of radius–ulna and tibia bones. After bleeding, the hot carcasses were properly handled following procedures described in Reference [22]. The slaughter liveweight (84 d, SLW) and hot carcass weight (HCW) were measured. The dressing out percentage (DoP) was defined as HCW divided by SLW.

Two meat quality traits include cooking loss (CL) and drip loss (DL). The drip loss was quantified by the method of Reference [23]. The cooking loss was measured throughout using the following method—approximately 20 g samples of cube-like raw meat from the biceps femoris muscle of the hind leg were weighed (W1) and steamed for 30 min. Cooked samples were cooled down to room temperature and re-weighed (W2). CL was calculated as follows:

CL (%) = 100 × (W2/W1)(1)

### 2.3. SLAF-Seq Design

The rabbit genome (GenBank assembly accession: GCA_000003625.1) was used as the reference genome to predict and characterize the presence of putative restriction endonuclease sites. Genome assembly revealed a genome size of 2.74 Gbp and a GC (guanine-cytosine) content of 43.75%. Restriction endonuclease profiles of the reference genome were determined using self-developed software [16]. The most efficient enzyme digestion scheme was selected based on the following criteria—(1) the proportion of restriction fragments located in the repetitive region is as low as possible; (2) zymogenic fragments are distributed evenly in the genome; (3) the number of observed restriction fragments (SLAF tags) meets the expected number of tags according to the primary in silico investigation based on reference genome sequences. 

Genomic DNA was extracted from whole blood samples using the phenol–chloroform method and was digested with the selected optimal enzymes. A single “A” nucleotide was added to the 3′ end of the digested fragment (SLAF tag). Dual-index [24] sequencing adapters were ligated to the A-tailed fragments using T4 DNA ligase. Further, PCR was conducted using diluted digested DNA samples, with forward primer (5′-AATGATACGGCGACCACCGA-3′) and reverse primer (5′-CAAGCAGAAGACGGCATACG-3′). PCR products were purified, pooled and separated by electrophoresis using a 2% agarose gel. Fragments of 300–500 bp size were excised and purified using a QIA Gel Extraction Kit (Qiagene, Germany). The library was sequenced on an Illumina HiSeq 2500 (Illumina, Inc., San Diego, CA, USA) platform following the manufacturer’s instructions. Raw sequencing reads were identified by dual-indexing and classified to each sample. Clean reads were mapped to the reference genome (GenBank assembly accession: GCA_000003625.1) using SOAP software (http://soap.genomics.org.cn) [25]. The sequences mapped to the reference genome were retained for further analysis.

### 2.4. Genotyping and Statistical Analysis

Efficiency of RsaI EcoRV-HF^®^ digestion was evaluated using a positive control sample (*Oryza sativa* ssp. *japonica*). SLAF tags were mapped to the reference genome using BWA software (http://bio-bwa.sourceforge.net/) [26] and SNPs were identified using two methods, GATK [27] and Samtools [28]. SNPs identified using both these methods with integrity > 0.8 and MAF > 0.05 were retained for GWAS analysis. 

The population structure of the rabbits was evaluated using the ADMIXTURE program [29]. The association analysis between traits and SNPs was performed according to a general linear mixed (GLM) model using PLINK2 software (http://www.cog-genomics.org/plink/2.0/) (--glm) [30,31]. The SNP effects were estimated using the following model:

(2) y=Xb+Zu+e

where y is the vector of phenotypes; b is the fixed effect of sex; u is the SNP effect with $u\;\sim \;N\left(0,\;I\sigma_{u}^{2}\right)$; X and Z are each the incidence matrix for b and u, respectively; and e is a vector of residual effects with $e\;\sim \;N\left(0,\;I\sigma_{e}^{2}\right)$. I is an identity matrix; $\sigma_{u}^{2}$ is the variance of SNP effects; and $\sigma_{e}^{2}$ is the residual variance.

Furthermore, Bonferroni correction was applied to determine significance at the genome-wide level. SNPs with an adjusted *p*-value less than the 10%genome-wide Bonferroni-corrected threshold were annotated using ANNOVAR software [32]. Genome-wide linkage disequilibrium (LD) blocks were estimated using PLINK2 (--ld) and the LD decay is shown in Supplementary Figure S1. Based on the LD decay, the genes within 100 kb of significant associated SNPs were considered as trait-associated candidate genes. We extracted the candidate genes within the ±100 kb region of the associated SNPs according to the genome annotation information in GFF format using a script written in-house in Python3 programming language.

## 3. Results and Discussion

### 3.1. Phenotype

Bodyweight at six time points, two carcass traits and two meat quality traits were measured. The descriptive statistics for the measured phenotypic traits are listed in Table 1.

### 3.2. SLAF-seq 

**Figure 1:**
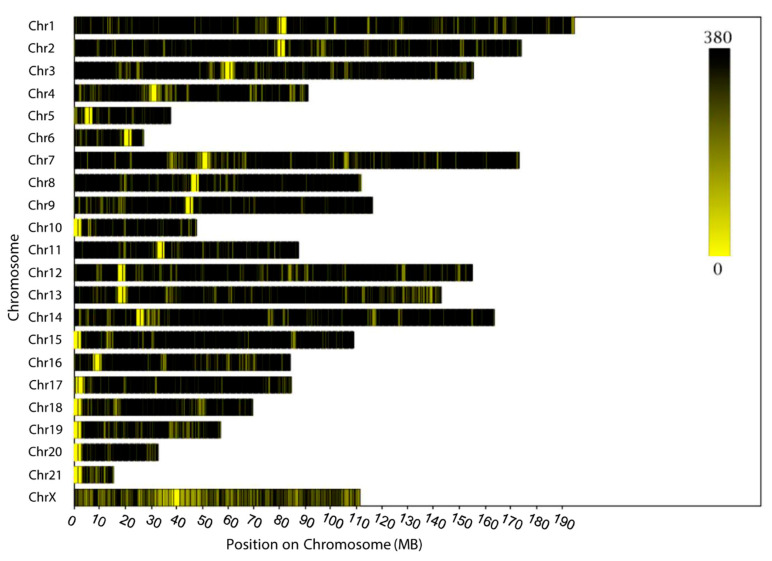
Single-nucleotide polymorphism (SNP) density distribution on chromosomes of the rabbit genome. The horizontal axis (*X*-axis) shows the chromosome length (Mbp). SNP density was calculated per 1 Mbp window. Different colors represent different SNP density levels.

A total of 3,127,736,005 paired-end reads was generated in this study. Almost 97.07% of the raw reads were successfully mapped to the rabbit genome. The effectiveness of digestion is the key indicator of a successful SLAF-seq. In this study, 96.05% of the sequences were digested normally, which is an ideal digestion rate. A total of 5,223,720 SLAF tags was identified across the whole genome, with sequencing to a 16.57× average depth. A total of 2,498,314 polymorphic SLAF tags was identified and 8,838,009 SNPs were selected for genome-wide association analysis based on the selection criteria (integrity > 0.8; minor allele frequency > 0.05). The density distribution of SNPs was calculated throughout the rabbit genome and is shown in Figure 1. Almost all of the genome’s non-overlapped 1 Mbp region contained SNPs, which indicates that the data are reliable.

### 3.3. Population Structure Analysis

**Figure 2:**
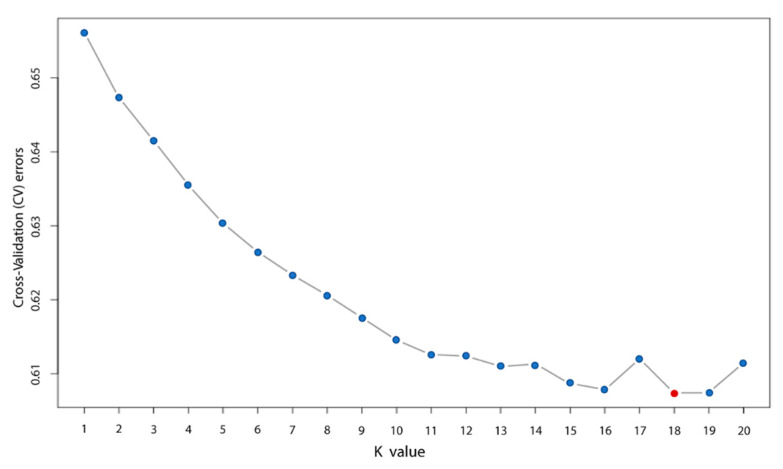
The coefficient of variation for each K value. The point of the lowest cross-validation (CV) error is indicated with red color.

The population structure of the rabbits was analyzed using the ADMIXTURE program. The population was first divided into 1–20 subgroups (K). The cross-validation (CV) error of populations was calculated under different k numbers. The number of the Kvalue with the lowest CV is the most suitable. Our analysis revealed that a K value of 18 was most optimal. The samples were divided into 18 subgroups (Figure 2). 

### 3.4. Genome-Wide Association Analysis

As population stratification might affect GWAS, quantile–quantile (Q-Q) plots of all traits were drawn. The observed *p* value calculated by the association study fit the expected ones, which suggests that the population stratification was well-corrected and the association analysis using GLM was reliable. The Q-Q plot of each trait is shown following the Manhattan plot of the corresponding trait.

GWAS based on SLAF-seq were successfully used to identify SNPs associated with important economic traits in chickens and pigs [18,19]. In this study, we performed SLAF-seq-based GWAS of six growth, two carcass and two meat quality traits using a General Linear Mixed (GLM) Model in meat rabbits. Table 2 shows the number of SNPs associated with each trait and the number of genes located within 100 kb upstream and downstream. Table S1 shows the location on the genome, the *p* value and the annotation of all SNPs linked with each trait.

**Figure 3:**
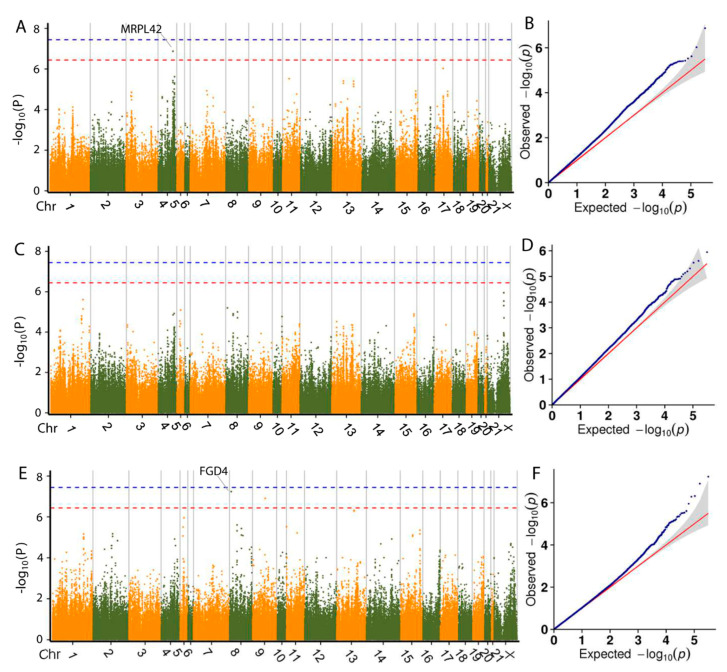
Manhattan plots and quantile–quantile (Q-Q) plots of the general linear mixed (GLM)-based genome-wide association study (GWAS) for bodyweight at day 35 (**A**,**B**), 42 (**C**,**D**) and 49 (**E**,**F**). Negative log_10_ *p* values of the filtered high-quality SNPs were plotted against their genomic positions. The dashed lines of orange and blue indicate a 10% and 1% genome-wide Bonferroni-corrected threshold, respectively.

**Figure 4:**
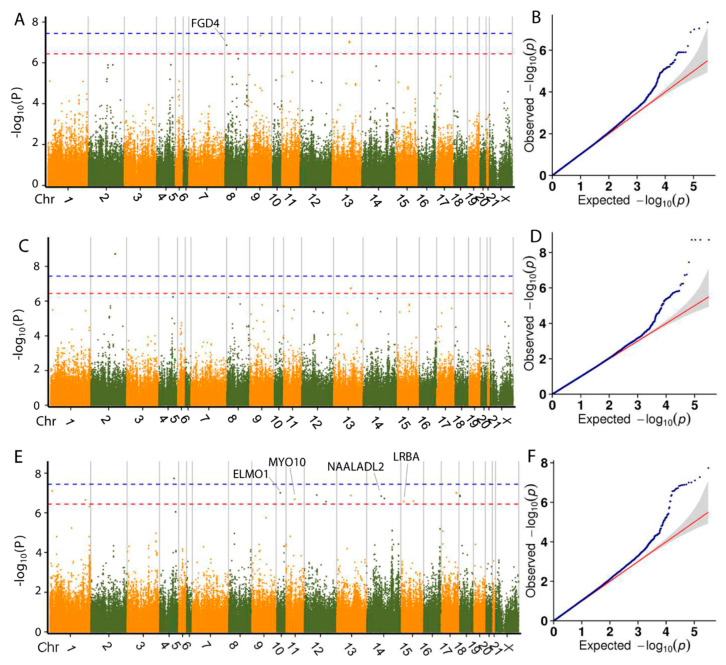
Manhattan plots and Q-Q plots of the GLM-based GWAS for bodyweight at day 56 (**A**,**B**), day 63 (**C**,**D**) and day 70 (**E**,**F**). Negative log_10_ *p* values of the filtered high-quality SNPs were plotted against their genomic positions. The dashed lines of orange and blue indicate 10% and 1% genome-wide Bonferroni-corrected threshold, respectively.

Based on the GLM and Bonferroni correction, 28 SNPs exhibited genome-wide association with the bodyweight trait at 35, 49, 56, 63 and 70 d (Table 2) and 16 genes near or within those SNPs were identified as associated with bodyweight. Manhattan plots for the growth traits are shown in Figure 3 and Figure 4. Interestingly, one SNP on chromosome 8 (Chr8) was found to be associated with bodyweight at both day 49 and 56, which is located within *FGD4* (FYVE, RhoGEF and PH domain containing 4). This gene encodes a protein involved in the regulation of actin cytoskeleton and cell shape [33], which is important for cell growth. The *DNM1L* (dynamin 1 like) gene, located within 100 kb of an SNP significantly associated with BW49 and BW56, encodes a member of the dynamin GTPase superfamily, which is involved in regulating mitochondrial metabolism. The gene expression of *DNM1L* is reported to increase in skeletal muscle following exercise [34]. We thus propose these genes as potential targets for molecular breeding in meat rabbits.

One SNP at position 61,268,427 of Chr9 was identified to be associated with bodyweight at day 49, 56 and 63. The SNP at position 8,681,239 in Chr8 was identified to be associated with bodyweight at day 49 and 56. SNPs at 83,815,488 and 83,815,516 of Chr13 were found to be associated with bodyweight at day 56 and 63. However, no genes were found within 100 kb upstream or downstream of these SNPs. We speculate these SNPs to have an association at two or more time points of bodyweight and be important for the growth of the rabbit and are potential targets for further molecular breeding research.

**Figure 5:**
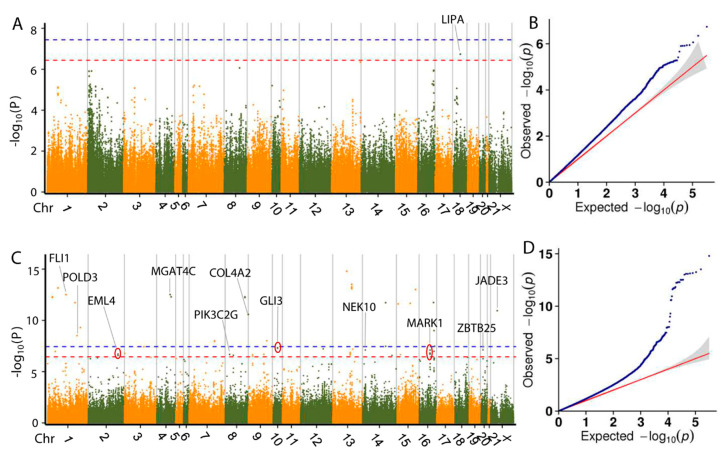
Manhattan plots and Q-Q plots of GLM-based GWAS for the dressing out percentage (**A**,**B**) and slaughter liveweight (**C**,**D**). Negative log_10_ *p* values of the filtered high-quality SNPs were plotted against their genomic positions. The dashed lines of orange and blue indicate a 10% and 1% genome-wide Bonferroni-corrected threshold, respectively. Red ellipses indicate that SNPs within the same ellipse share the nearest candidate gene.

A total of 81 SNPs was found to be associated with two carcass traits and 71 genes were found near those SNPs. Manhattan plots for the growth traits are shown in Figure 5. Among them, one SNP located at 37,208,481 of Chr18 was identified to be associated with slaughter liveweight (84 d), with six genes located around the SNP. We identified *LIPA* (lipase A, lysosomal acid type) as the nearest gene, which encodes lipase A, the lysosomal acid lipase (cholesterol ester hydrolase). This enzyme catalyzes the hydrolysis of cholesteryl esters and triglycerides in the lysosome [35]. In addition, 80 SNPs were found associated with carcass rate and 65 genes near these SNPs encoded proteins with various functions (Supplementary Material Table S1). However, there have been no reports of the association between these candidate genes and animal production traits. Further study is required to validate the links between candidate genes and traits.

**Figure 6:**
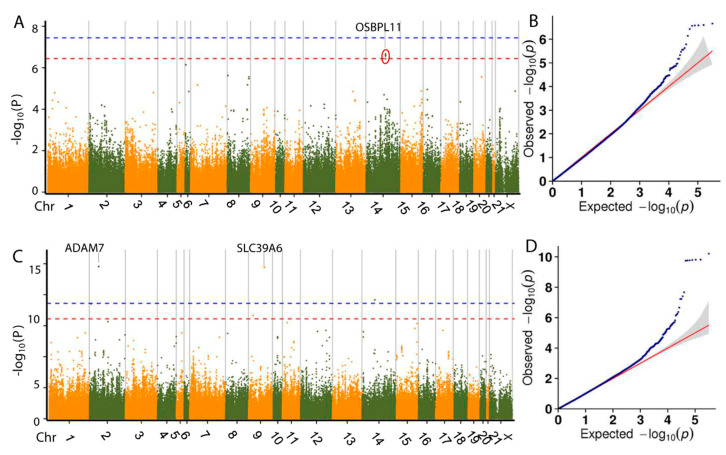
Manhattan plots and Q-Q plots of the GLM-based GWAS for the meat quality traits of cooking loss (**A**,**B**) and drip loss (**C**,**D**) in rabbits. Negative log_10_ *p* values of the filtered high-quality SNPs were plotted against their genomic positions. The dashed lines of orange and blue indicate a 10% and 1% genome-wide Bonferroni-corrected threshold, respectively. The red ellipse indicates that SNPs in the same ellipse share the nearest candidate gene.

For the meat quality phenotype, 15 SNPs were associated with cooking loss and drip loss. Manhattan plots for the growth traits are shown in Figure 6. Five SNPs were associated with cooking loss, with 2 genes located nearby. The nearest gene *OSBPL11* (oxysterol-binding protein like 11) encodes a member of the oxysterol-binding protein (*OSBP*) family, a group of intracellular lipid receptors [36]. Ten SNPs were associated with drip loss, with 9 genes around those SNPs. Two main types of proteins in the human body involved in the maintenance of zinc ion homeostasis—zinc binding protein, which acts as a buffer substance or as a donor of intracellular zinc and zinc transporters, which are involved in the intake and excretion of zinc in cells. Among the five genes found in close proximity to the associated SNPs of the drip loss trait in this study, two encode proteins involved in the maintenance of zinc homeostasis. *ADAM7* (ADAM metallopeptidase domain 7) on chromosome 2 encodes a member of the *ADAMs* family of zinc proteases and *SLC39A6* (solute carrier family 39 member 6) on chromosome 9 encodes a protein with structural characteristics of zinc transporters [37]. These findings indicate that the potential targets for meat quality are the genes relevant to zinc homeostasis. 

## 4. Conclusions

In this study, we performed GWAS of 432 F1 meat rabbits. All samples were genotyped using SLAF-seq technology. The GWAS of 10 economic traits revealed 111 significantly associated SNPs. A total of 98 putative candidate genes were located within 100 kb of significantly associated SNPs. Among them, *FGD4* and *DNM1L* genes, linked with BW49 and BW56 in our study, are reported to be involved in cell growth and mitochondrial metabolism. *ADAM7* on chromosome 2 and *SLC39A6* on chromosome 9 may be associated with drip loss due to its effect on zinc homeostasis that functions in determining meat quality. These findings provide novel insights into the genetic basis of growth, carcass and meat quality traits in rabbits and may contribute to the application of GS in rabbits. However, because only a draft version of the rabbit reference genome is available, there are numerous sequence gaps and missing annotations. These gaps could influence the accurate estimation of LD patterns. Moreover, the missing annotations would inevitably lead to false-negative results when attempting to find causative genes or variants. In addition, relatively high numbers of false positives were observed in the GWAS study. Therefore, a future validation study should be conducted using an independent population.

## Figures and Tables

**Table 1:** Descriptive statistics of phenotypic traits.

Traits	Acronym	Trait Group	N	Mean	SD
35-day BW, g	BW35	Growth	432	787.4213	129.6323
42-day BW, g	BW42	Growth	423	1007.229	124.2199
49-day BW, g	BW49	Growth	419	1234.916	155.4796
56-day BW, g	BW56	Growth	412	1460.34	211.3732
63-day BW, g	BW63	Growth	392	1686.722	279.0648
70-day BW, g	BW70	Growth	382	1927.864	320.2864
Slaughter liveweight (84 d), g	SLW	Carcass	432	2082.037	313.2049
Dressing out percentage, %	DoP	Carcass	430	48.7838	3.9789
Cooking loss, %	CL	Meat quality	432	68.1618	5.383
Drip loss, %	DL	Meat quality	432	4.7754	1.6808

BW: bodyweight; SD: standard deviation.

**Table 2:** Number of SNPs associated with each trait and the number of genes around.

Trait Group	Traits	# SNP	# Gene
growth	35 d BW	1	4
growth	42 d BW	0	0
growth	49 d BW	2	3
growth	56 d BW	4	3
growth	63 d BW	7	N/A
growth	70 d BW	19	9
carcass	Slaughter liveweight (84 d)	1	6
carcass	Dressing out percentage	80	65
meat quality	Cooking loss	5	2
meat quality	Drip loss	10	9

## Supplementary Materials

The following are available online at https://www.mdpi.com/2076-2615/10/6/1068/s1. Figure S1. A schematic representation of genome-wide linkage disequilibrium (LD) decay. Table S1. Significant SNPs for growth, carcass and meat quality traits in rabbits.
