# Supplementary figures and images for: A Genome-Wide Association Study Identifying Genetic Variants Associated with Growth, Carcass and Meat Quality Traits in Rabbits
Source: Animals (Basel). 2020 Jun 20;10(6):1068. doi: 10.3390/ani10061068 (PMC7341332; doi:10.3390/ani10061068)

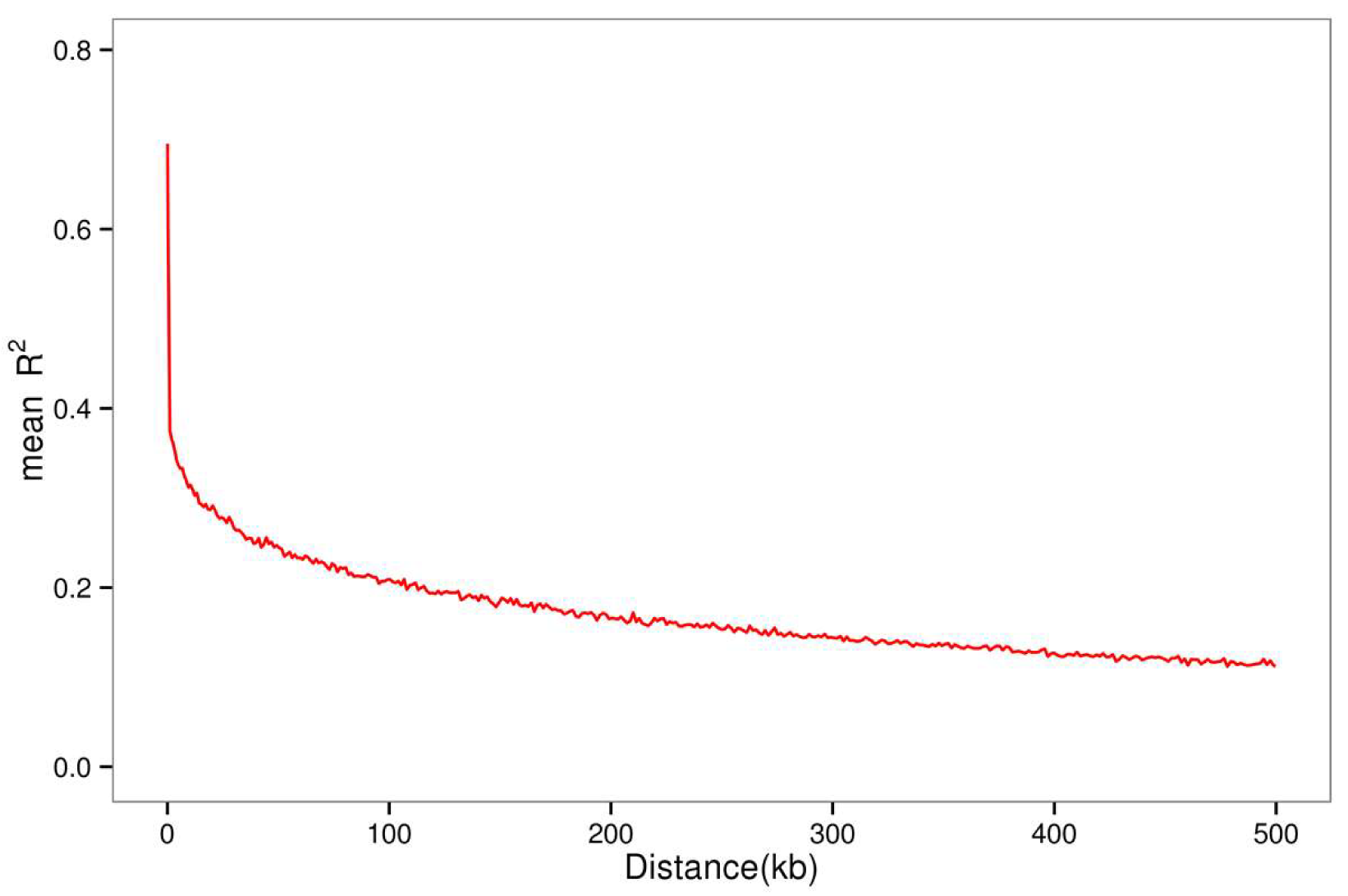

Supplement: Supplementary file 1 [file animals-10-01068-s001.zip › Supplementary/Figure S1 LD decay.tif]
